# The Future Entrance Standard

**Published:** 1898-06

**Authors:** 


### Editorial.

### THE FUTURE ENTRANCE STANDARD.

THE period is approaching when the question of entrance standard to our dental colleges must seriously be considered by the National Association of Dental Faculties; and while it is not the purpose of this article to forestall opinion or to influence minds, doubtless confirmed in the views entertained by the majority at the last meeting of that body, it is necessary that the thought of dental educators should be turned in this direction that they may be prepared to act intelligently when gathered together at Omaha.

The Association of Faculties was subjected to severe criticism for its action at Old Point Comfort by a certain class, whose knowledge of educational matters has been limited to their connection with boards of examiners. Unfortunately the mass of the dental profession take the verdict of these men as representing the highest intelligent opinion upon the subject, and treat the action of the faculties and the reasons given for this with undisguised contempt.

The decision of that association has passed into history, and there is no disposition to traverse that further than to repeat in substance the statement of the editorial in the October number of this journal, that the Association of Faculties did the only thing possible under the circumstances and, for the time, adopted the right course of action.

The growth of communities in intelligence and prosperity cannot be accomplished by forceful measures, but must be left to the slow evolutionary advances of time. This is so much of a truism that it remains a matter of constant astonishment that men seem to fail to recognize it as a law of the universe. As well attempt to make a mature human being of an infant as to force society, institutions, or peoples to an advanced stage of intellectual growth by process of law. This fact being recognized, it becomes our duty to plan the development of our profession upon principles in harmony with it, and avoid the unpleasant experience, so frequently witnessed, of being obliged to retrace the steps taken, a result to be deplored, as it retards the accomplishment of the object sought to be attained.

The arguments brought forward to sustain a forced advance are based mainly on two propositions: first, that a better element is needed in dentistry; and, second, that the dental profession is overcrowded and means must be instituted to lessen the increasing numbers.

The first proposition is conceded. In all callings the demand is for greater intelligence, a demand never satisfied, and which probably will never be so long as mentality is working upward towards the greatest possible attainment,—the goal of perfection. This aim simply represents the evolutionary force which has brought about the product which we call civilization, and which must continue to be exerted as long as the race continues to exist.

The second proposition, that the dental profession is overcrowded, would mean simply a reiteration of statements based on statistics. Taking this view, it possibly is overcrowded, if the number in practice be compared with population, and yet even here there is a very respectable clientage for every man and woman engaged in the practice of dentistry. It is forgotten, it is presumed, that the colleges are not only educating these to practise, but they are at the same time educating the masses to a correct appreciation of dentistry as a means of prolonging life and deliverance from suffering. This education of the people is rapidly developing beyond the bounds of mere luxury to that of an urgent necessity. Every year, therefore, adds to the demand for skilled laborers in the field, and this must continue as intelligence increases.

Having conceded the importance of a higher standard for entrance to the dental profession, it becomes essential that the further question must be considered. How far can this go without injury to the practical side of dentistry? It has been recognized by all writers upon dental work that the foundation of this calling was built in this country by men whose attainments were largely mechanical. It is further recognized that had it been placed in the hands of graduates of literary colleges with the medical degree superadded, it would have proved in practical excellence a decided failure. It is unnecessary to refer here to instances of this in its history. Dentistry has been in the past strictly a mechanical calling with a thin veneer of culture added. It has been the aim of later years to reduce the former and increase the latter, until the veneering has been transferred to the preliminary education, and the presumably solid theoretical training placed in the closing years. This means, as it seems to the writer, a deterioration in the not far-distant future in all that appertains to good work.

It is difficult to draw a correct dividing line between mechanical training, on the one hand, and culture, on the other, both being essential to ultimate success; but, if one is to be sacrificed, it would seem to be better to give more time to the former than to the latter, as this will ordinarily result in the greatest benefit to the patient and operator.

It is just here that the increase in the standard for entrance to our dental colleges must be considered. If we are to train men to practical excellence, and this is certainly the paramount object, and at the same time give character to our calling, there must be a reasonable division made between practice and culture. Extremes in either direction will result in incalculable damage to dentistry, and may retard its healthy growth through generations. Every step taken must therefore be weighed in the interest of all concerned.

The Association of Faculties very wisely hesitated to change the existing order until time had been given for calm reflection. This time has now arrived, and it is for this body at its next meeting to consider and to *act*.

The conditions existing in the United States are not those of older countries, and any action that fails to take these into consideration will result in serious failure. Aside from these, we are brought face to face with a practical difficulty that, in the view of the writer, is of vital importance. It has been proposed in some quarters that a standard equivalent to a diploma from a high school should be required for entrance to dental colleges. This has been insisted upon as a requirement by at least one State board. This must be regarded as a very high standard. If universally adopted, it would entirely debar a very worthy class of young men hampered by limited means from ever being able to practise in its ranks. To this the selfish side of dentistry would answer, "This is exactly what we desire." The better and altruistic side would reply, "As dentistry has grown in the past through the efforts of this class, can it justly deprive it of its rightful inheritance, and would it not in the end insure its own destruction?"

The college side of the question can take care of itself. It is not of so much importance that all of these institutions should be sustained, while the adoption of any standard which would result in the destruction of those at present established upon a good working basis would be regarded as a calamity.

The question of greatest importance seems to pivot round the idea, "Is it possible to advance the standard to that named and now in force in several dental institutions? What are the conditions?" The average age of entrance to a high school is, it is assumed, about sixteen years. The diploma cannot be earned in the best institutions short of three years, and generally four. This brings the proposed student to twenty. His mind is stored with a large amount of digested and undigested knowledge, but his fingers are, as a rule, utterly untrained in the proper movement of anything, not even the pen, if we may judge by the writing of many of them. It is a recognized fact in all trades that a man cannot acquire these unless he starts not later than sixteen, and no employer would undertake to train a person in mechanical dexterity at twenty or twenty-one. While there are exceptions to this, these will be found possessing that natural bent that leads some to mechanics in spite of their surroundings, but these need not be considered in this inquiry. It has been the observation of all teachers placed in close touch with dental students that those who enter at the advanced age mentioned are slow to acquire mechanical dexterity, and the majority fail to excell. In proportion as years go by this ability to acquire is decreased. So true is this that the conscientious dean will discourage the man of thirty-five from attempting it, unless he be fortified by previous practice in other lines of mechanical work.

While this statement has been antagonized in certain quarters it remains with the writer a fact of observation, and should be considered in any attempt to advance the standard.

If, then, this be admitted, an effort should be made to regulate the standard to bring the proposed matriculation not later than eighteen years and not higher than is possible of attainment by any energetic person in or out of the high school. Changes in the standard must recognize the possibilities of being able to work up to it by those outside of such schools. The effort to reach the standard of a high-school diploma would be beyond the possibility of any except the most determined and free from pecuniary embarrassments.

What, then, should be the course of action in the Association of Faculties? To the reasonable thinking mind this should not at present go beyond the first year of a high school. Let this be the standard now, and it can be left for time to solve the problem of a still further advance. The experiment is now being tried by the departments of several of the universities and one or two isolated schools. This is as it should be, and no criticism can be made against this action. A few years will demonstrate the effect upon those adopting this high standard; but, whether for good or ill to these, their action should not be taken as the standard for the entire educational body. This would, beyond all question, be disastrous to dentistry, to the colleges, and, in a broader sense, to the humanitarian aims of the dental profession.

